# Structural and magnetic investigations of the Weyl semimetal family CeAlSi_1–*x*_Ge_*x*_

**DOI:** 10.1107/S2052520626002635

**Published:** 2026-04-07

**Authors:** Daniel A. Mayoh, Sanjay Sharma, Thomas W. Chamberlain, George D. A. Wood, Ivan da-Silva, Paul A. Goddard, Martin R. Lees, Geetha Balakrishnan

**Affiliations:** a Physics Department, University of Warwick, Coventry, CV4 7AL, United Kingdom; b ISIS Neutron and Muon Source, Rutherford Appleton Laboratory, Didcot, OX11 0QX, United Kingdom; SIMaP, France

**Keywords:** Weyl semimetal, topological magnetism, crystal structure, crystal growth

## Abstract

We report a systematic study of the structural and magnetic evolution in CeAlSi_1–*x*_Ge_*x*_, a series of materials which provide a tunable platform for exploring magnetism in noncentrosymmetric Ce-based intermetallics. Polycrystalline samples and single crystals were synthesized using arc melting and flux growth techniques. Structural characterization by X-ray diffraction shows a continuous increase in the unit-cell parameters with increasing Ge content, with no evidence of a structural phase transition across the series. Magnetization measurements reveal a suppression of the ferromagnetic ordering temperature of CeAlSi with increasing Ge substitution, indicating a crossover toward antiferromagnetic behaviour in Ge-rich compositions. Neutron diffraction measurements performed on selected compositions show that weak magnetic intensity appears on some structural Bragg peaks below the magnetic ordering temperature. These results elucidate the relationship between chemical substitution, crystal structure, and magnetic ground states in CeAlSi_1–*x*_Ge_*x*_, and establish this system as a model platform for studying compositionally tuned magnetic order in noncentrosymmetric materials.

## Abstract 2

A systematic investigation is reported of the structural and magnetic evolution in the CeAlSi_1–*x*_Ge_*x*_ series, combining X-ray and neutron diffraction with magnetization measurements on polycrystalline samples and single crystals. Chemical substitution leads to a continuous lattice expansion and a suppression of the ferromagnetic ordering temperature without a structural phase transition, revealing a crossover towards antiferromagnetic behaviour and establishing CeAlSi_1–*x*_Ge_*x*_ as a model system for compositionally tuned magnetism in noncentrosymmetric materials.

## 1. Introduction

Weyl semimetals are a novel class of three-dimensional matter where, in the bulk, the conduction and valence bands cross at discrete points known as Weyl nodes (Xu *et al.*, 2015 ▸). These Weyl nodes manifest themselves in the form of exotic surface states called Fermi arcs and through emergent electromagnetic responses, such as the anomalous Hall effect and the topological Hall effect (Armitage *et al.*, 2018 ▸; Yang *et al.*, 2018 ▸; Gooth *et al.*, 2017 ▸; Wawrzik *et al.*, 2021 ▸). Magnetic Weyl semimetals hold the promise of exhibiting topological states that can be easily tuned by the application of small magnetic fields (Yan & Felser, 2017 ▸). Weyl semimetals can be either type-I, having standard Weyl points with a point-like Fermi surface, or type-II, where the Weyl point is still a protected crossing but appears at the contact of electron and hole pockets. They can be generated by either breaking inversion symmetry, such as in nonmagnetic TaAs (Lv *et al.*, 2015 ▸), or time-reversal symmetry, as in ferromagnetic Weyl semimetals, such as Co_3_Sn_2_S_2_ (Morali *et al.*, 2019 ▸) and Co_2_MnGa (Swekis *et al.*, 2021 ▸).

**Figure 1:**
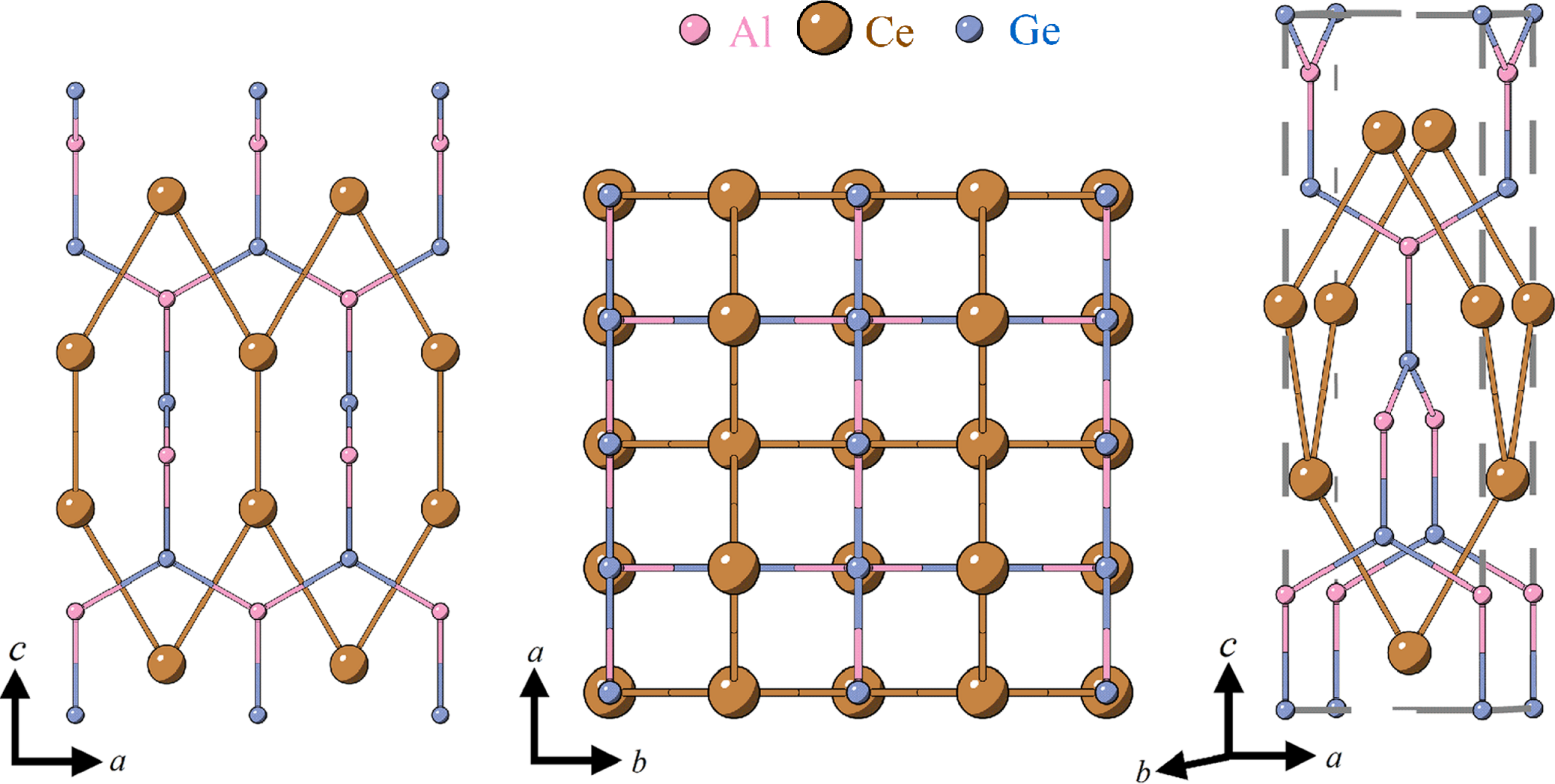
Crystal structure of CeAl*X* in the noncentrosymmetric space group *I*4_1_*md* shown along several crystallographic directions. The Ce and Al atoms are indicated in brown and pink, respectively. The *X* atoms, which may be Si, Ge, or a mixture of both, are indicated in blue. The dashed lines indicate the unit cell of CeAl*X*.

CeAl*X* (where *X* = Si, Ge) are a family of magnetic Weyl semimetals that has generated significant attention due to the high tunability of their magnetic properties (Cheng *et al.*, 2024 ▸; Li *et al.*, 2023 ▸). This tunability arises from several degrees of freedom, the first being that the rare-earth and Al–*X* stoichiometry are predicted to control the number and location of Weyl nodes, as well as the type of broken symmetry (Yan & Felser, 2017 ▸). Furthermore, CeAl*X* has been reported to crystallize in either the noncentrosymmetric *I*4_1_*md* (Singh & Mukherjee, 2020 ▸) or the centrosymmetric *I*4_1_*amd* space groups. (Bobev *et al.*, 2005 ▸) The noncentrosymmetric crystal structure of CeAl*X* is shown in Fig. 1 ▸.

In addition to forming inversion-breaking crystal structures, CeAlSi and CeAlGe possess magnetically ordered ground states and therefore also break time-reversal symmetry.

CeAlGe was initially reported to be ferromagnetic (FM) with *T*_c_ = 5.6 K (Flandorfer *et al.*, 1998 ▸). More recent studies show CeAlGe exhibits a complicated antiferromagnetic (AFM) incommensurate multi-*k* structure below *T*_N_ = 4.4 K in zero field and a field-induced topological state (Puphal *et al.*, 2019 ▸; Puphal *et al.*, 2020 ▸). The magnetic response is anisotropic with an in-plane (*H* ∥ *a*) Curie–Weiss temperature, θ_CW_, of −42 K and an out-of-plane (*H* ∥ *c*) θ_CW_ = 10 K (Puphal *et al.*, 2019 ▸). Below *T*_N_, the low-field dc magnetic susceptibility is an order of magnitude larger for *H* ∥ *a* than for *H* ∥ *c* (Hodovanets *et al.*, 2018 ▸).

Unlike other ferromagnetic Weyl semimetals which typically show collinear FM ordering, CeAlSi exhibits a net magnetization along the crystallographic [110] direction and a spin texture below *T*_c_ = 8.2 (3) K, described by the authors as a noncollinear FM (Yang *et al.*, 2021 ▸). CeAlSi displays two different anomalous Hall effects depending on whether a magnetic field is applied along an easy axis (in the *ab* plane) or a hard axis (out-of-plane) (Yang *et al.*, 2021 ▸). The magnetic response of CeAlSi is also anisotropic. Below *T*_C_, the low-field dc magnetic susceptibility is over 50 times higher for *H* ∥ *a* than for *H* ∥ *c* (Yang *et al.*, 2021 ▸). Polycrystalline CeAlSi is reported to have a θ_CW_ = −25.5 K (Dhar & Pattalwar, 1996 ▸), despite ordering ferromagnetically. There are no reports for the magnetic susceptibility of single crystal CeAlSi in the paramagnetic state.

Here, we present a study of the structural and magnetic properties of the CeAlSi_1–*x*_Ge_*x*_ family of materials. In particular, we focus on the crossover between the ferromagnetic CeAlSi and antiferromagnetic CeAlGe in both polycrystalline samples and single crystals, to determine how this crossover manifests. We use a combination of powder X-ray diffraction (PXRD), powder neutron diffraction (PND), and magnetization measurements to study changes in both the crystallographic and magnetic properties as Si is replaced with Ge.

## 2. Experimental

Polycrystalline materials were synthesized by arc melting. Crystal growths were carried out using the flux method with Al flux. All sample preparation techniques are discussed in more detail in the following section. The crystal structure and phase purity of the samples were determined using powder X-ray diffraction, which was carried out using a Malvern Panalytical Empyrean diffractometer equipped with a Cu tube (λ = 1.5418 Å) and an Anton Paar XRDynamic 500 diffractometer equipped with a Co target (λ = 1.7902 Å). A Co source was used to minimize the fluorescence arising from Ce in the samples. Low-temperature powder X-ray diffraction was carried out using a Panalytical Empyrean X-ray diffractometer equipped with a Mo source (λ = 0.7107 Å) and an Oxford Cryosystems PheniX sample chamber capable of reaching temperatures down to 12 K under vacuum. Rietveld refinements were carried out on the observed diffraction patterns using the *GSAS-II* software suite (Toby & Von Dreele, 2013 ▸) and the *FULLPROF* software suite (Rodriguez-Carvajal *et al.*, 2025 ▸). Powder neutron diffraction experiments on selected samples were carried out on the GEM diffractometer at the ISIS neutron source, STFC, UK (Chamberlain *et al.*, 2023 ▸).

A Laue camera system (Photonic Science) was used to collect back-scattered X-ray Laue diffraction patterns of crystals to assess crystal quality and crystallographic alignment. Energy-dispersive X-ray (EDX) analysis measurements were performed using a Zeiss Supra 55-VP FEGSEM to check the stoichiometry and identify any elemental variations across the samples. The magnetic properties of the polycrystalline materials were measured using a Quantum Design Magnetic Property Measurement System. Temperature-dependent dc magnetic susceptibility (χ) measurements were carried out over the temperature range 1.8–300 K in applied magnetic fields of up to 50 kOe.

## 3. Crystal growth and sample synthesis

Samples were prepared from Ce (ingot, Sigma-Aldrich, 99.9%), Al (shot, Sigma-Aldrich, 99.9%), Si (granules, NewMet, 99.9999%), and Ge (ingot, ABCR, 99.9999%). Surface oxides were removed from the rare-earth metals by polishing prior to weighing. The Al was cleaned in a dilute HCl solution.

### 3.1. Polycrystalline samples

Polycrystalline samples of CeAlSi_1–*x*_Ge_*x*_, with nominal *x* = 0.0, 0.3, 0.4, 0.5, 0.6, 0.7 and 1.0, were synthesized by arc melting stoichiometric amounts of the cleaned elements in an arc furnace under an argon atmosphere. The resulting sample buttons were flipped and remelted three times to ensure good homogeneity. The observed weight loss during melting was negligible in all cases.

### 3.2. Single-crystal growth

Single crystals of the CeAl*X* compounds were grown using the self-flux method. The starting materials Ce, Al, and (Si/Ge) were mixed in a ratio of 1:20:1 and placed into a Canfield crucible with 5 ml capacity cylindrical crucibles (ACP-CCS-5, LSP Industrial Ceramics, inc.). The crucible was then sealed in a quartz ampoule under vacuum. The ampoule was heated to 1223 K and held for 2 h before being cooled at a rate of 2.5 K h^−1^ to 958 K. The ampoule was then removed from the furnace and immediately centrifuged to remove the excess Al flux. Any remaining flux was subsequently removed using a dilute NaOH solution.

**Figure 2:**
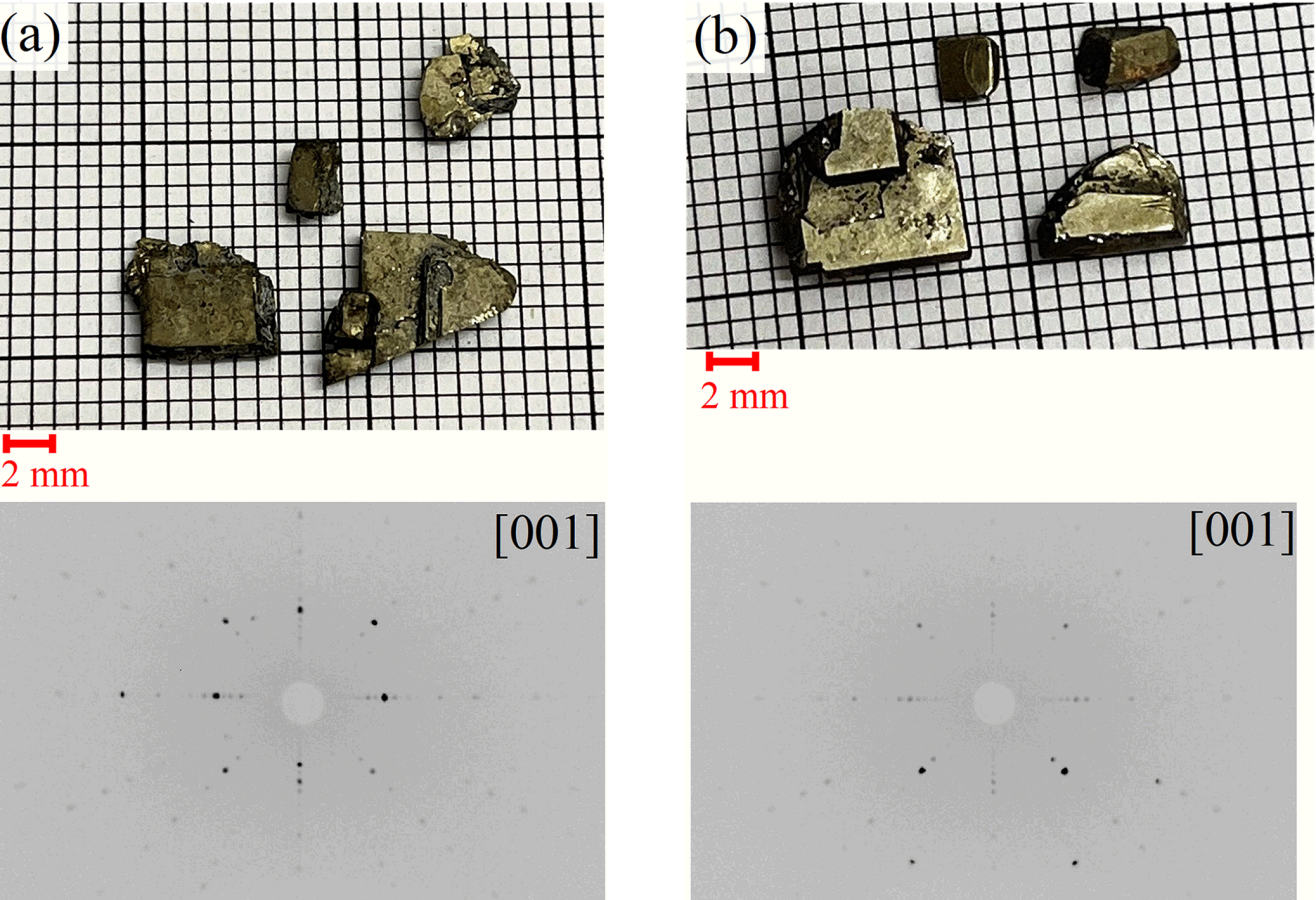
Crystals of (*a*) CeAlSi and (*b*) CeAlGe grown using the flux method. Below each image is the corresponding Laue diffraction pattern obtained by back-scattering X-rays from the largest crystal face. The observed fourfold symmetry indicates that the diffraction pattern corresponds to the [001] crystallographic direction, confirming that the largest face is perpendicular to the *c* axis of the crystal.

The flux growths of all the CeAl*X* compounds yielded large, square-faceted crystals with approximate dimensions of 5 mm × 5 mm × 2 mm, with the largest faces lying in the *ab* plane, as shown in Fig. 2 ▸. It was noted, particularly in the case of CeAlGe, that the crystal surfaces tarnished relatively quickly under ambient conditions due to oxidization.

## 4. Results and discussion

### 4.1. Powder X-ray diffraction

**Figure 3:**
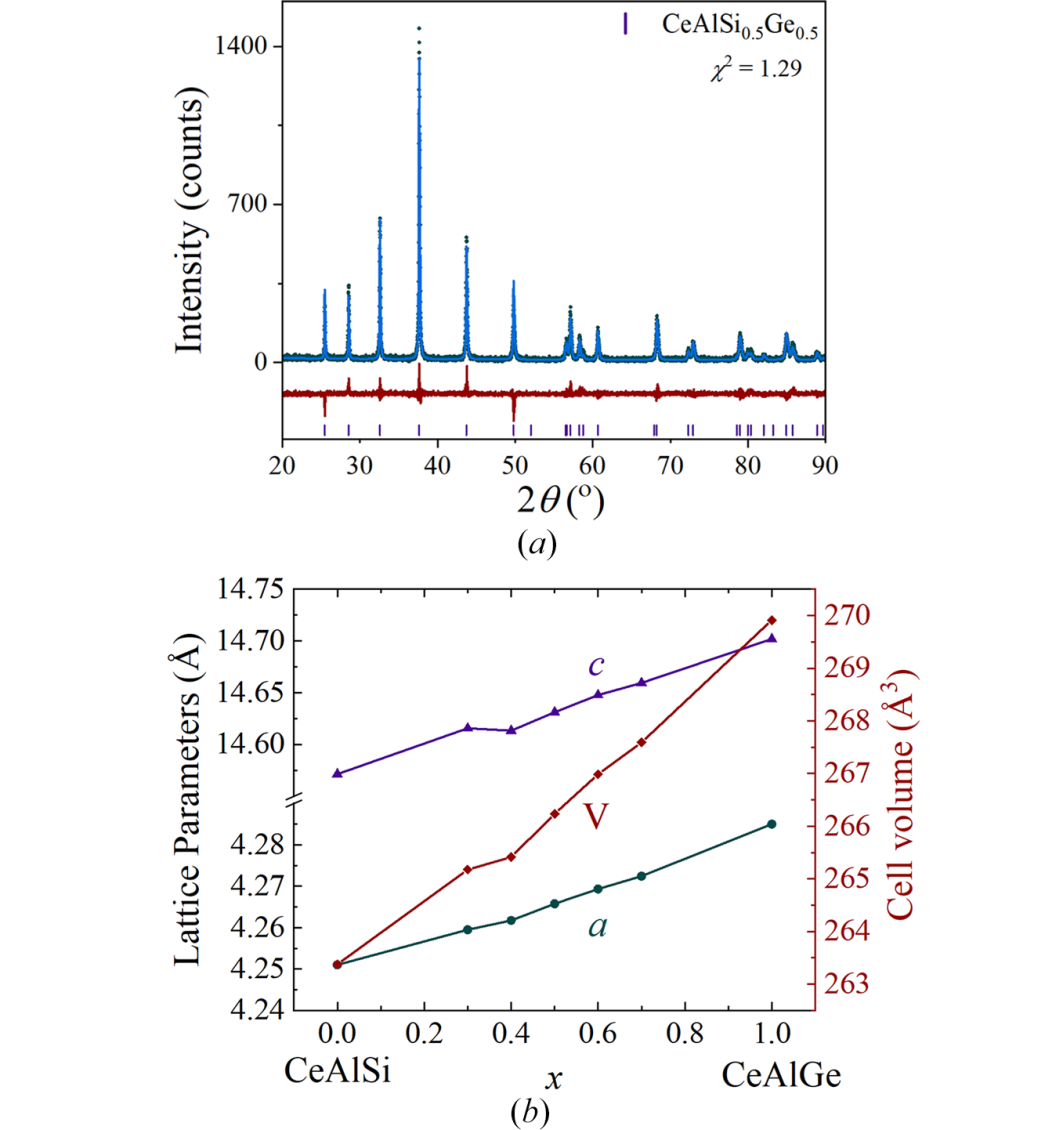
(*a*) Room-temperature powder X-ray diffraction pattern of a CeAlSi_0.5_Ge_0.5_ collected with a Co target (λ = 1.7902 Å). The experimental profile (green circles), the Rietveld refinement of the data (blue line) and the difference between the two (red line) are shown. Also shown are the expected Bragg peaks indicated by the purple vertical lines. (*b*) Unit-cell parameters *a* and *c*, and unit-cell volume *V*, as a function of Ge concentration *x* for CeAlSi_1–*x*_Ge_*x*_.

Structural analysis of the synthesized polycrystalline CeAlSi_1–*x*_Ge_*x*_ samples with nominal composition *x* = 0.0, 0.3, 0.4, 0.5, 0.6, 0.7 and 1.0, was performed using powder X-ray diffraction collected at room temperature. The powder X-ray diffraction pattern of CeAlSi_0.5_Ge_0.5_ is shown in Fig. 3 ▸(*a*). Rietveld refinements were carried out to determine the phase purity and unit-cell parameters for each compound, which are shown in Table 1 ▸. These values are in agreement with previous reports (Pang *et al.*, 2022 ▸). They demonstrate that, as the Si at the 4*a* site is substituted with Ge, an expansion of the unit-cell parameters and unit-cell volume is observed, as expected.

**Figure 4:**
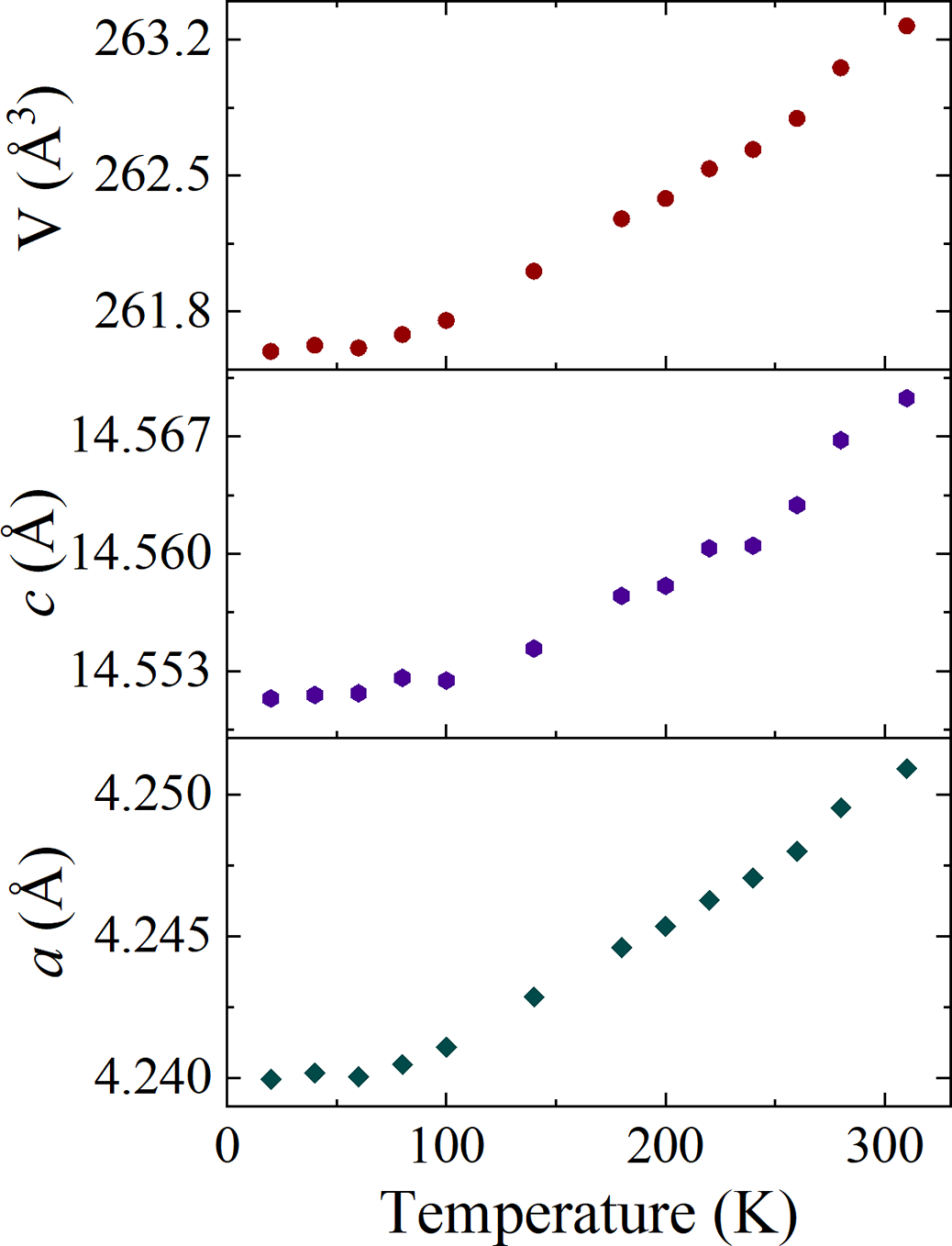
Unit-cell parameters *a* and *c*, and the unit-cell volume *V*, of CeAlSi as a function of temperature from 20 to 310 K determined from PXRD. A decrease in *a*, *c*, and *V* is observed with decreasing temperature, and there is no indication of any abrupt change in the unit-cell parameters that would suggest a structural phase transition.

**Figure 5:**
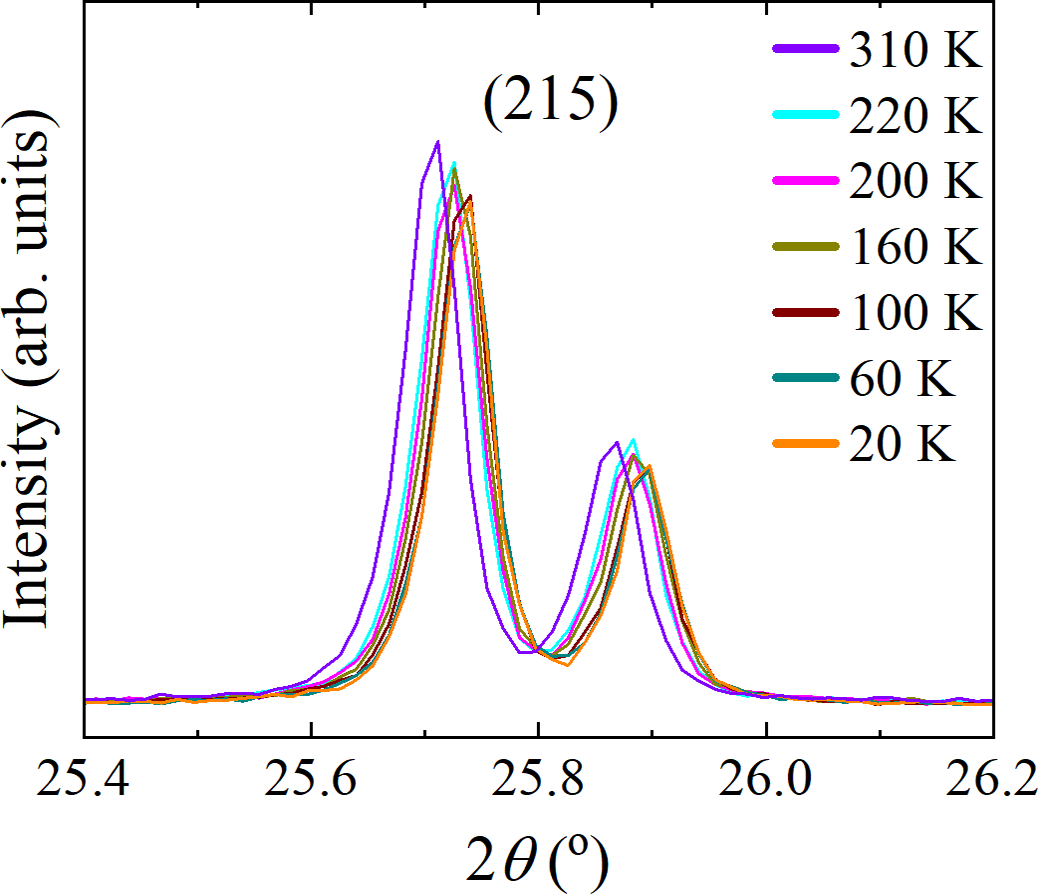
Powder X-ray diffraction data for CeAlSi collected with a Mo source (λ_*K*α1_ = 0.7093 and λ_*K*α2_ = 0.7135 Å) at selected temperatures from 20 to 310 K, highlighting the shift in the (215) peak.

In an effort to probe any low-temperature structural transitions in CeAlSi, PXRD patterns were collected at 20 K intervals while cooling from 300 to 20 K. Finally, the sample was heated to 310 K and a pattern was collected to check that the change in the unit-cell parameters is reversible. Rietveld refinements were performed on each of the collected patterns. Changes in unit-cell parameters and unit-cell volumes as a function of temperature, are shown in Fig. 4 ▸. The unit-cell parameters decrease with temperature and no obvious abrupt changes were observed. This suggests that no structural transitions occur between 310 and 20 K in this material. It was observed that upon cooling, the Bragg peaks exhibited a gradual shift in peak position due to the temperature-dependent unit-cell contraction. Fig. 5 ▸ shows a selected 2θ range highlighting the shift in the (215) peak.

### 4.2. Compositional analysis

The elemental compositions of the polycrystalline CeAlSi_1–*x*_Ge_*x*_ materials were measured using energy-dispersive X-ray analysis. Estimates of the relative Ce, Al, Si, and Ge content of the polycrystalline samples are given in Table 2 ▸. The results show that the Si:Ge ratios in each of the materials are within ±10% of the expected values.

### 4.3. Magnetic susceptibility

**Figure 6:**
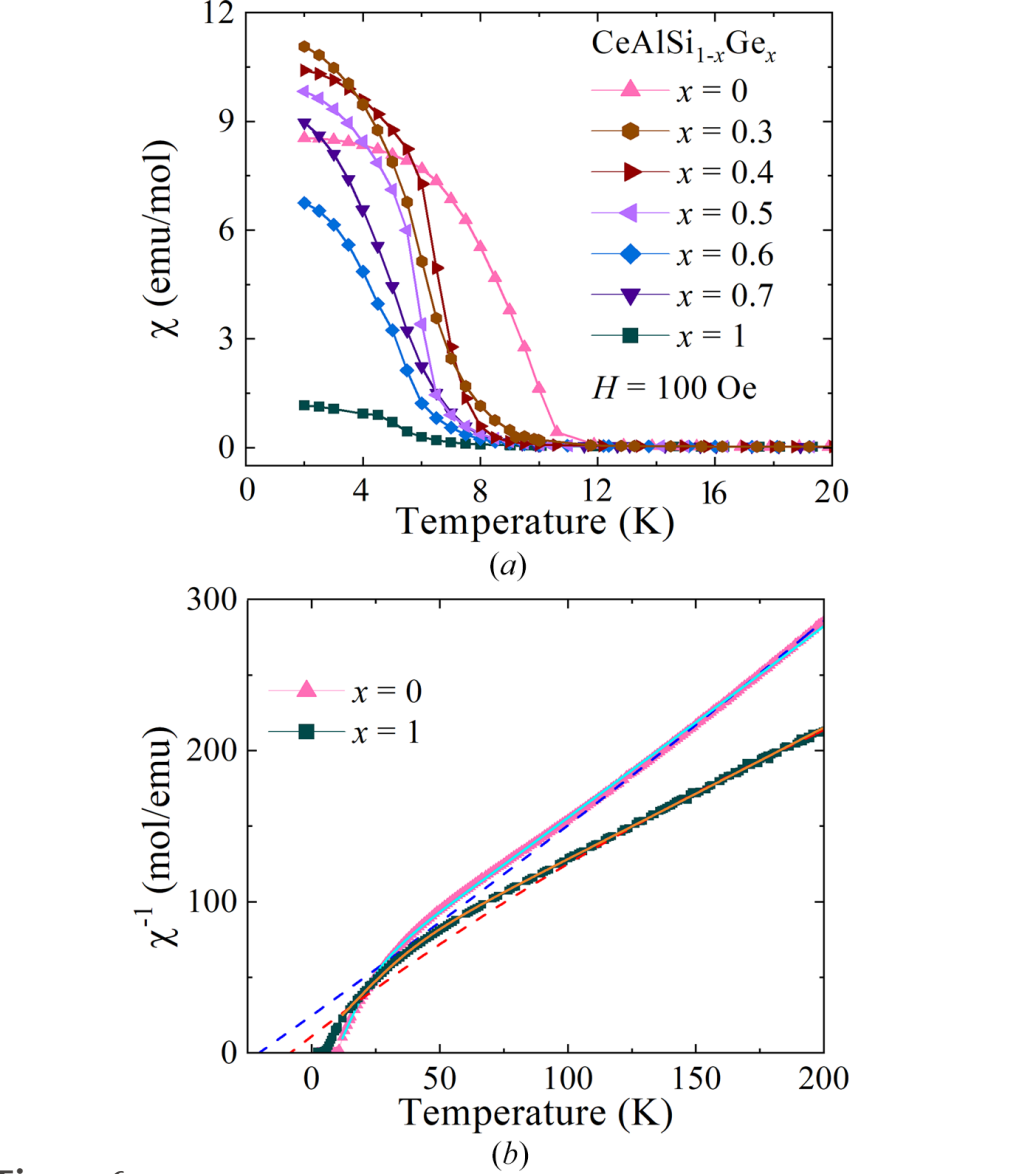
(*a*) Temperature dependence of the dc magnetic susceptibility χ(*T*) for polycrystalline samples of CeAlSi_1–*x*_Ge_*x*_ collected in an applied magnetic field of 100 Oe in field-cooled cooling mode. (*b*) Inverse magnetic susceptibility versus temperature for polycrystalline CeAlSi and CeAlGe. Fits to a modified Curie–Weiss law and a two-level model (see text) are shown by dashed and solid lines, respectively.

Magnetic susceptibility versus temperature data were collected for the polycrystalline CeAlSi_1–*x*_Ge_*x*_ samples in an applied field of 100 Oe in field-cooled cooling (f.c.c.) mode (see Fig. 6 ▸). For materials with 0.3 ≤ *x* ≤ 0.7 the temperature dependence and magnitude of χ(*T*) are similar to that of CeAlSi, while χ(*T*) for CeAlGe is an order of magnitude smaller. This provides evidence that the crossover to AFM order occurs at a Ge content of *x* > 0.7. The magnetic ordering temperature, *T*_c_, determined from the maximum in |*d*χ(*T*)/*dT*|, is 9.9 (5) K for CeAlSi. This falls to 6.3 (5) K for *x* = 0.3 followed by an almost linear decrease thereafter across the series (see Table 3 ▸). These observations are consistent with previous reports (Suzuki *et al.*, 2019 ▸; Puphal *et al.*, 2019 ▸).

χ(*T*) between 150 and 300 K in the paramagnetic state were fit to a modified Curie–Weiss expression, χ(*T*) = *C*/(*T* − θ_CW_) + χ_0_, where χ_0_ accounts for the van Vleck magnetism, diamagnetic contributions from the ion cores, and any signal from the sample holder. The Curie–Weiss temperatures, θ_CW_, are all negative [see Fig. 6 ▸(*b*) and Table 3 ▸]. The results agree well with previous work for polycrystalline CeAlSi [−25.5 K; (Dhar & Pattalwar, 1996 ▸)] and CeAlGe [−18 K; Flandorfer *et al.*, 1998 ▸), −13.5 K (Dhar & Pattalwar, 1996 ▸)]. The effective moments extracted from the Curie constant, *C*, all lie in the range 2.45–2.8 μ_B_, close to that expected value for trivalent free ion Ce^3+^ (2.54 μ_B_). Note, however, the values of *C*, θ_CW_, and χ_0_ determined from these fits are coupled, and depend strongly on the temperature range of the fitting. An observed curvature in χ^−1^(*T*) at lower temperatures arises from the effects of crystalline electric fields. The data can be fit to a two-level model (Mitric *et al.*, 1997 ▸)
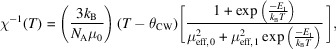
where *k*_B_ is the Boltzmann constant, *N*_A_ is Avogadro’s number, μ_0_ is the permeability of free space, *E*_1_ is the energy splitting to the first excited crystal field level with an effective moment of 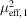, while 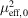 is the effective moment of the crystal field ground state. μ_eff_ = μ_B_g_*J*_[*J*(*J* + 1)]^1/2^, where *g*_*J*_ is the Landé *g*-factor and *J* is the total angular momentum quantum number. Fitting gives a θ_CW_ for CeAlSi that is clearly positive, while θ_CW_ for samples with 0.3 ≤ *x* < 1 are all close to zero, and θ_CW_ = −2.2 (4) K for CeAlGe (see Table 3 ▸). At room temperature the calculated effective moments all lie in the range 2.4–3.0 μ_B_, while at low temperatures the effective moments are reduced, consistent with a single Kramers doublet with effective spin 1/2 as the ground state (Yang *et al.*, 2021 ▸).

**Figure 7:**
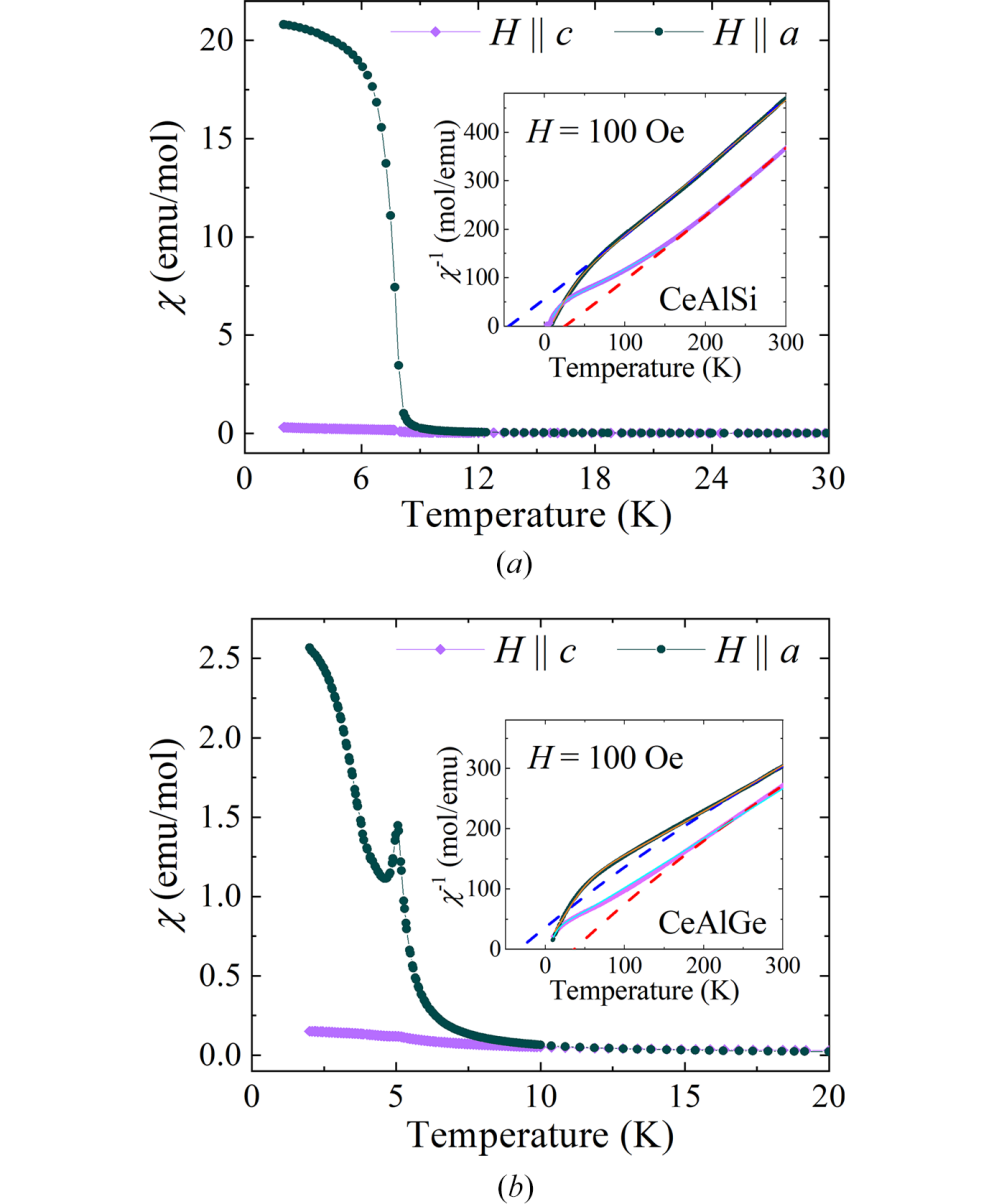
Magnetic susceptibility as a function of temperature for (*a*) CeAlGe and (*b*) CeAlSi measured in an applied field of 100 Oe along two crystallographic directions. No observable difference was found between zero-field-cooled and field-cooled data; therefore only the field-cooled data are shown for clarity. The insets show the inverse magnetic susceptibility as a function of temperature, together with fits to a modified Curie–Weiss model limited to higher temperatures (*T* > 200 K) and two-level model down to 10 K.

Magnetic susceptibility versus temperature data were also collected on single crystals of CeAlSi and CeAlGe, with a magnetic field of 100 Oe applied along the *a* and *c* axes. The field-cooled cooling curves are shown in Fig. 7 ▸. The ordering temperature for single crystal CeAlSi is slightly lower than the equivalent polycrystalline material. For CeAlGe, a peak in χ(*T*) may indicate that the transition to the magnetic ground state proceeds via an intermediate phase (Hodovanets *et al.*, 2018 ▸). In both samples, the magnetic response is highly anisotropic. Below the magnetic ordering temperature, χ(*T*) is significantly larger for *H* ∥ *a*. The inverse susceptibility as a function of temperature [shown in the insets of Figs. 7 ▸(*a*) and 7 ▸(*b*)] reveal a crossover, with the susceptibility at room temperature larger for *H* ∥ *c* in both compounds. Curie–Weiss fits made above 200 K, where χ^−1^(*T*) are almost linear, yield effective magnetic moments of 2.5–2.68 μ_B_. The Curie–Weiss temperatures for both CeAlSi and CeAlGe are positive for *H* ∥ *c* and negative for *H* ∥ *a* (Puphal *et al.*, 2019 ▸). These observations are consistent with the values for the polycrystalline samples, assuming χ_poly_ = (2χ_*a*_ + χ_*c*_)/3.

Fits using a two-level model produce similar values for the effective moments, however, the θ_CW_ temperatures are positive for CeAlSi and negative for CeAlGe, for both field directions (see Table 3 ▸). These observations underline the importance of strong crystal electric field anisotropy and exchange interactions in determining the magnetic properties of these CeAlSi_1–*x*_Ge_*x*_ materials (Jin *et al.*, 2025 ▸).

### 4.4. Neutron diffraction

**Figure 8:**
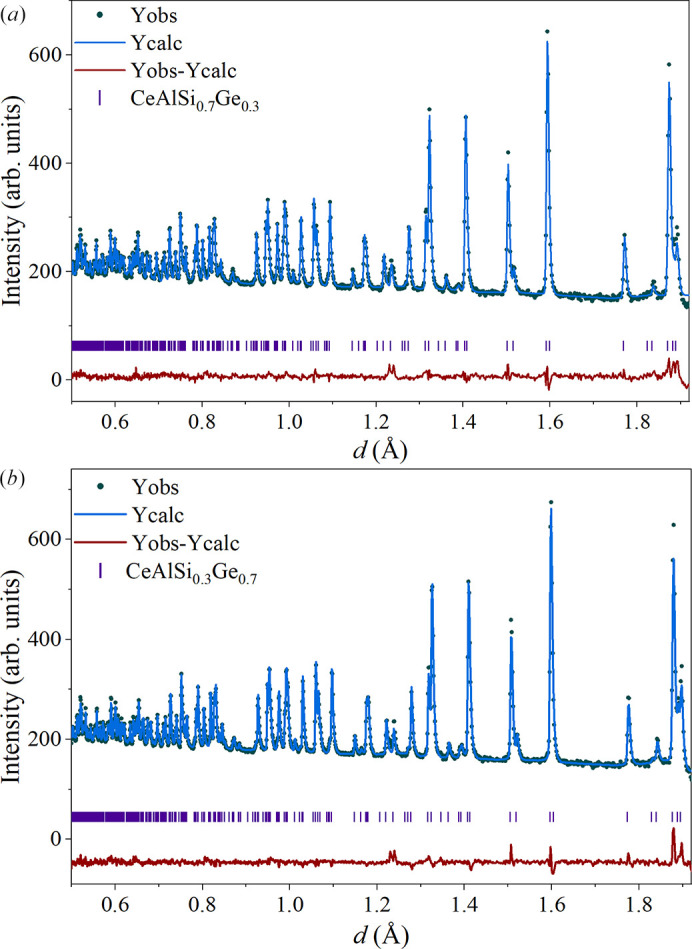
Powder neutron diffraction data and Rietveld refinements of the nuclear structures of (*a*) CeAlSi_0.7_Ge_0.3_ and (*b*) CeAlSi_0.3_Ge_0.7_ at 1.7 K. The observed, calculated, and difference patterns are shown as green points, blue lines, and red lines, respectively. Nuclear Bragg peak positions are indicated by purple "|" tick marks.

Powder neutron diffraction data for CeAlSi_0.7_Ge_0.3_ and CeAlSi_0.3_Ge_0.7_ were obtained using the GEM spectrometer at ISIS, STFC. The nuclear structure for both compounds was determined from the Rietveld refinements of the powder neutron diffraction data collected at 1.7 K, as shown in Fig. 8 ▸. The unit-cell parameters, occupation numbers and atomic positions determined from these refinements are given in Table 4 ▸. These values are consistent with both our powder X-ray diffraction data (see Table 1 ▸) and previous reports (Pang *et al.*, 2022 ▸). No noticeable impurities were observed in either data set.

**Figure 9:**
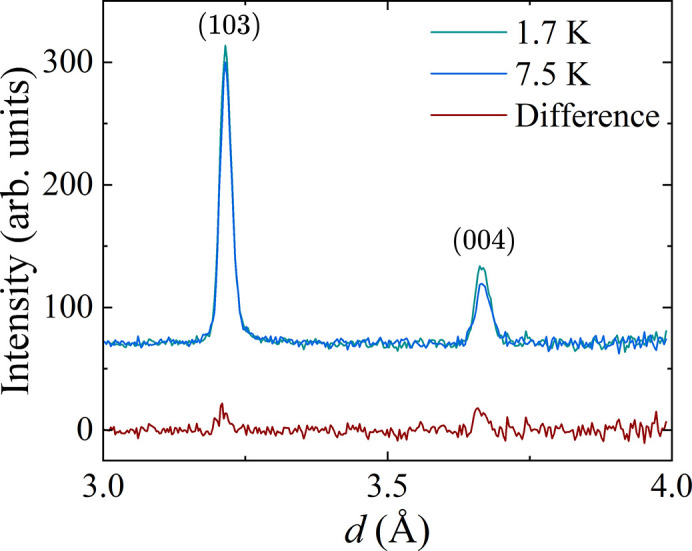
Powder neutron diffraction data for CeAlSi_0.3_Ge_0.7_ at 1.7 and 7.5 K showing a change in intensity of the (103) and (004) peaks consistent with the onset of long-range magnetic order.

In order to investigate the magnetic ordering exhibited by CeAlSi_0.3_Ge_0.7_, neutron diffraction data were also collected at 7.5 K (just above the expected magnetic transition temperature) and compared with the data collected at 1.7 K. Cooling the samples from 7.5 to 1.7 K resulted in the appearance of additional intensity that is magnetic in origin in two of the Bragg peaks, as shown in Fig. 9 ▸. The magnetic peaks are coincident with the nuclear peaks, indicating *k* = 0 ordering. Due to the poor statistics and limited number of magnetic peaks, it was not possible to determine the magnetic structure from these data. Weak magnetic peaks have been observed in neutron scattering data for the end-member compounds CeAlGe (Suzuki *et al.*, 2019 ▸; Puphal *et al.*, 2020 ▸; Pomjakushin *et al.*, 2025 ▸) and CeAlSi (Yang *et al.*, 2021 ▸) and the magnetic structures reported, however, there are no reports as yet, of the magnetic structure determined by neutron scattering in these substituted materials.

## 5. Conclusions

The family of Weyl semimetals CeAlSi_1–*x*_Ge_*x*_ (where *x* = 0.0, 0.3, 0.4, 0.5, 0.6, 0.7 and 1.0) has been investigated in both polycrystalline and single-crystal form. Polycrystalline powders of CeAlSi_1–*x*_Ge_*x*_ were synthesized by arc melting. The effect of substituting Ge on the Si site was examined using powder X-ray diffraction, which revealed an expansion of the unit-cell parameters with no observable change in the crystal structure. DC magnetic susceptibility measurements show a decrease in the magnetic ordering temperature as the Si is progressively replaced with Ge. Powder neutron diffraction data collected on CeAlSi_0.7_Ge_0.3_ and CeAlSi_0.3_Ge_0.7_ show a nuclear structure consistent with the tetragonal *I*4_1_*md* space group, with weak magnetic peaks observed below the magnetic transition temperature of CeAlSi_0.3_Ge_0.7_.

## Supplementary Material

Crystal structure: contains datablock(s) global, CeAlSi, CeAlSi0.3Ge0.7, CeAlSi0.4Ge0.6, CeAlSi0.5Ge0.5, CeAlSi0.6Ge0.4, CeAlSi0.7Ge0.3, CeAlGe, CeAlSi0.3Ge0.7neutron, CeAlSi0.7Ge0.3neutron. DOI: 10.1107/S2052520626002635/vel5001sup1.cif

Rietveld powder data: contains datablock(s) CeAlSi. DOI: 10.1107/S2052520626002635/vel5001CeAlSisup2.rtv

Rietveld powder data: contains datablock(s) CeAlSi0.3Ge0.7. DOI: 10.1107/S2052520626002635/vel5001CeAlSi0.3Ge0.7sup3.rtv

Rietveld powder data: contains datablock(s) CeAlSi0.4Ge0.6. DOI: 10.1107/S2052520626002635/vel5001CeAlSi0.4Ge0.6sup4.rtv

Rietveld powder data: contains datablock(s) CeAlSi0.5Ge0.5. DOI: 10.1107/S2052520626002635/vel5001CeAlSi0.5Ge0.5sup5.rtv

Rietveld powder data: contains datablock(s) CeAlSi0.6Ge0.4. DOI: 10.1107/S2052520626002635/vel5001CeAlSi0.6Ge0.4sup6.rtv

Rietveld powder data: contains datablock(s) CeAlSi0.7Ge0.3. DOI: 10.1107/S2052520626002635/vel5001CeAlSi0.7Ge0.3sup7.rtv

Rietveld powder data: contains datablock(s) CeAlGe. DOI: 10.1107/S2052520626002635/vel5001CeAlGesup8.rtv

CCDC references: 2546564, 2546565, 2546566, 2546567, 2546568, 2546569, 2546570, 2546571, 2546572

## Figures and Tables

**Table 1:** Parameters determined from refinement of powder X-ray data collected at room temperature

Composition	Space group	χ^2^	GOF	*a* (Å)	*c* (Å)	Atom	Wyckoff position	*x*	*y*	*z*	Occupancy
CeAlSi	*I*4_1_*md*	3.22	1.79	4.2514 (6)	14.5712 (3)	Ce	4*a*	0	0	0.5764 (7)	0.998 (2)
						Al	4*a*	0	0	0.1651 (10)	1.11 (3)
						Si	4*a*	0	0	0	1.03 (3)
CeAlSi_0.7_Ge_0.3_	*I*4_1_*md*	1.26	1.10	4.2594 (2)	14.6155 (7)	Ce	4*a*	0	0	0.58 (5)	0.995 (9)
						Al	4*a*	0	0	0.16 (7)	1.01 (8)
						Ge	4*a*	0	0	0	0.29 (8)
						Si	4*a*	0	0	0	0.70 (9)
CeAlSi_0.6_Ge_0.4_	*I*4_1_*md*	1.51	1.23	4.2625 (2)	14.6177 (1)	Ce	4*a*	0	0	0.574 (1)	0.92 (1)
						Al	4*a*	0	0	0.163 (2)	1.03 (9)
						Ge	4*a*	0	0	0	0.39 (2)
						Si	4*a*	0	0	0	0.59 (7)
CeAlSi_0.5_Ge_0.5_	*I*4_1_*md*	1.29	1.11	4.2657 (7)	14.6311 (3)	Ce	4*a*	0	0	0.5752 (5)	0.954 (6)
						Al	4*a*	0	0	0.1673 (18)	0.94 (8)
						Ge	4*a*	0	0	0	0.511 (7)
						Si	4*a*	0	0	0	0.500 (8)
CeAlSi_0.4_Ge_0.6_	*I*4_1_*md*	1.21	1.11	4.2693 (1)	14.6477 (4)	Ce	4*a*	0	0	0.5755 (9)	0.98 (2)
						Al	4*a*	0	0	0.1661 (12)	1.07 (9)
						Ge	4*a*	0	0	0	0.53 (8)
						Si	4*a*	0	0	0	0.42 (12)
CeAlSi_0.3_Ge_0.7_	*I*4_1_*md*	1.37	1.17	4.2724 (3)	14.6594 (9)	Ce	4*a*	0	0	0.568 (2)	0.93 (2)
						Al	4*a*	0	0	0.158 (2)	1.05 (6)
						Ge	4*a*	0	0	0	0.68 (8)
						Si	4*a*	0	0	0	0.31 (5)
CeAlGe	*I*4_1_*md*	2.28	1.51	4.2847 (4)	14.7018 (2)	Ce	4*a*	0	0	0.5783 (3)	0.99 (1)
						Al	4*a*	0	0	0.1707 (8)	0.89 (3)
						Ge	4*a*	0	0	0	0.97 (2)

**Table 2:** Elemental composition of CeAlSi_1–*x*_Ge_*x*_ samples with a nominal *x* = 0.0 0.3, 0.4, 0.5, 0.6, 0.7 and 1.0 determined from EDX analysis

Nominal composition	Ce	Al	Si	Ge
Polycrystalline				
CeAlSi	1.05 (1)	1.06 (1)	1.00 (1)	0.00
CeAlSi_0.7_Ge_0.3_	1.09 (1)	1.14 (1)	0.72 (1)	0.28 (1)
CeAlSi_0.6_Ge_0.4_	1.03 (1)	1.07 (1)	0.59 (1)	0.41 (1)
CeAlSi_0.5_Ge_0.5_	1.06 (1)	0.91 (1)	0.48 (1)	0.52 (1)
CeAlSi_0.4_Ge_0.6_	1.05 (1)	1.13 (1)	0.44 (1)	0.56 (1)
CeAlSi_0.3_Ge_0.7_	1.12 (1)	0.96 (1)	0.28 (1)	0.72 (1)
CeAlGe	1.06 (1)	0.97 (1)	0.00	1.00 (1)
Single crystal				
CeAlSi	0.99 (1)	1.02 (1)	1.03 (1)	0.00
CeAlGe	1.02 (1)	0.98 (1)	0.00	1.05 (1)

**Table 3:** Properties of CeAlSi_1–*x*_Ge_*x*_ determined from magnetic susceptibility data

		CW fits		Two-level fits		
*x*	*T*_c_ (K)	θ_CW_ (K)	μ_eff_ (μ_B_)	θ_CW_ (K)	μ_eff_ (μ_B_)	
Polycrystalline						
0	9.9 (5)	−21 (2)	2.59 (2)	9.4 (3)	2.38 (3)	
0.3	6.3 (5)	−41 (5)	2.58 (1)	−1.2 (9)	3.58 (3)	
0.4	6.3 (5)	−22 (1)	2.61 (1)	0.0 (6)	2.59 (2)	
0.5	6.0 (5)	−26 (2)	2.73 (3)	0.8 (9)	3.07 (3)	
0.6	5.2 (5)	−25 (3)	2.78 (3)	−0.9 (4)	2.85 (1)	
0.7	5.1 (5)	−13 (5)	2.59 (5)	1.1 (4)	2.83 (3)	
1	5.2 (5)	−9 (5)	2.45 (5)	−2.2 (4)	2.83 (2)	
Single crystal						
0	7.8 (5)	25.5 (3)	2.54 (2)	4.1 (3)	2.79 (3)	*H* ∥ *c*
0		−44.2 (4)	2.53 (2)	8.0 (2)	2.25 (2)	*H* ∥ *a*
1	5.2 (5)	36.4 (4)	2.55 (1)	−2.25 (5)	2.99 (3)	*H* ∥ *c*
1		−33.3 (5)	2.68 (5)	−1.94 (3)	2.83 (3)	*H* ∥ *a*

**Table 4:** Parameters determined from the Rietveld refinement of powder neutron diffraction collected at 1.7 K

Compounds	CeAlSi_0.7_Ge_0.3_	CeAlSi_0.3_Ge_0.7_
Space group	*I*4_1_*md*	*I*4_1_*md*
*a*, *b*, *c* (Å)	4.2493 (1), 4.2493 (1), 14.605 (2)	4.2591 (7), 4.2591 (7), 14.6400 (4)
*R*_p_	10.3	9.9
*R*_wp_	9.75	9.97
Ce		
*x*, *y*, *z*	0, 0, 0.5812 (5)	0, 0, 0.5822 (4)
Occupancy	0.251 (2)	0.237 (1)
*U*_iso_	0.13 (4)	0.15 (3)
Al		
*x*, *y*, *z*	0, 0, 0.1662 (2)	0, 0, 0.1663 (2)
Occupancy	0.255 (1)	0.254 (1)
*U*_iso_	0.45 (8)	0.67 (6)
Si		
*x*, *y*, *z*	0, 0, 0	0, 0, 0
Occupancy	0.176 (1)	0.175 (1)
*U*_iso_	0.013 (8)	0.48 (4)
Ge		
*x*, *y*, *z*	0, 0, 0	0, 0, 0
Occupancy	0.074 (2)	0.075 (2)
*U*_iso_	0.013 (8)	0.48 (4)

## Data Availability

Data is available on Warwick WRAP and ISIS DataGateway.
